# The effect of myocardial action potential duration on cardiac pumping efficacy: a computational study

**DOI:** 10.1186/s12938-018-0508-2

**Published:** 2018-06-15

**Authors:** Da Un Jeong, Ki Moo Lim

**Affiliations:** Department of IT Convergence Engineering, Kumoh National Institute of Technology, 61 Daehak-ro, Gumi, Gyeongbuk 39177 Republic of Korea

**Keywords:** Myocardial action potential, Action potential duration, I_Ks_ channel, Conductivity, Cardiac pumping, Arrhythmia, Computational simulation

## Abstract

**Background and aims:**

Although studies on the relation between arrhythmias and the action potential duration (APD) have been carried out, most of them are based only on electrophysiological factors of the heart and lack experiments that consider cardiac mechanical and electromechanical characteristics. Therefore, we conducted this study to clarify the relevance of the shortening of APD of a cell in relation to the mechanical contraction activity of the heart and the associated risk of arrhythmia.

**Methods:**

The human ventricular model used in this study has two dynamic characteristics: electrophysiological conduction and mechanical contraction. The model simulating electrophysiological characteristics was consisted of lumped parameter circuit that can mimic the phenomenon of ion exchange through the cell membrane of myocyte and consisted of 214,319 tetrahedral finite elements. In contrast, the model simulating mechanical contraction characteristics was constructed to mimic cardiac contraction by means of the crossbridge of a myofilament and consisted of 14,720 hermite-based finite elements to represent a natural 3D curve of the cardiac surface. First, we performed a single cell simulation and the electrophysiological simulation according to the change of the APD by changing the electrical conductivity of the *I*_*Ks*_ channel. Thus, we confirmed the correlation between APD and intracellular Ca^2+^ concentration. Then, we compared mechanical response through mechanical simulation using Ca^2+^ data from electrical simulation.

**Results:**

The APD and the sum of the intracellular Ca^2+^ concentrations showed a positive correlation. The shortened APD reduced the conduction wavelength of ventricular cells by shortening the plateau and early repolarization in myocardial cells. The decrease in APD reduced ventricular pumping efficiency by more than 60% as compared with the normal group (normal conditions). This change is caused by the decline of ventricular output owing to reduced ATP consumption during the crossbridge of myofilaments and decreased tension.

**Conclusion:**

The shortening of APD owing to increased electrical conductivity of a protein channel on myocardial cells likely decreases the wavelength and the pumping efficiency of the ventricles. Additionally, it may increase tissue sensitivity to ventricular fibrillation, including reentry, and cause symptoms such as dyspnea and dizziness.

## Background

The heart’s blood pumping activity is caused by the repeated contraction and relaxation of the cardiac muscle. Contractions are caused by electrical conduction/propagation and the mechanical behavior of myocardial cells [1]. The action potential (AP) of ventricular myocardial cells leads to the contraction of myofilaments through the transmembrane passage of various electrical ions (especially Ca^2+^ ions) [2]. The AP may cause secretion or excretion of these ions, as well as myocardial contractility. Changes in AP may cause abnormal transmembrane currents in myocardial cells, which in turn may cause cardiac arrhythmias and other heart diseases.

There are many potential cause for membrane current abnormalities in myocardial cells. For example, abnormality of the protein channels inside the cell membrane directly affects the movement of ions entering and exiting the cell, thereby changing the action potential duration (APD) [3]. This is because the K^+^ channel plays an important role in determining the repolarization of the cell’s AP. Therefore, both the gain and loss of the K^+^ channel function can induce arrhythmia by affecting the APD [4, 5]. If the APD is reduced owing to changes in the K^+^ channel, the conduction wavelength of the heart is shortened, and the reentry can be easily induced. Conversely, prolonged APD can induce torsades de pointes tachycardia, leading to cardiac death [5, 6].

Clinical studies in 2008 discussed this phenomenon. According to Ravens et al., the higher the membrane conductivity on the K^+^ channel, the shorter the APD, facilitating the generation of reentrant waves [5]. It has been suggested that changes in APD may play a role in arrhythmogenesis and myocardial disease [7]. Animal experiment in 2017 demonstrated a linear correlation between myocardial contraction and APD, owing to the relationship between APD and ATP conductivity in K^+^ channels [8]. In addition, a computer simulation study showed that K^+^ channel abnormalities can affect arrhythmogenesis by predicting changes in APD at the single-cell level according to gain and loss of the K^+^ channel function [4].

These studies observed the electrophysiological effects of APD on the K^+^ channel at the single cell level, not in three-dimensional tissue. However, it is critical to observe such electrophysiological phenomena and the mechanical behavior of the heart in three-dimensional tissue, because the electrical stimulus for the ventricles in heterogeneously transferred from cell to cell or tissue to tissue. Furthermore, to thoroughly understand cardiac activity, it is necessary to conduct quantitative analysis of the electromechanical behavior of the heart in three-dimensional space.

We recently developed image-based three-dimensional ventricular electromechanical models, that can quantitatively compare the energy consumed by electrophysiological phenomena during mechanical beating [9–11]. In the present study we observed changes in APD according to the electrical conductivity of the K^+^ channel at the single-cell level, and extended these results to three-dimensional ventricular tissue.

## Methods

### Model of cellular electrophysiology and crossbridge dynamics

**Fig. 1:**
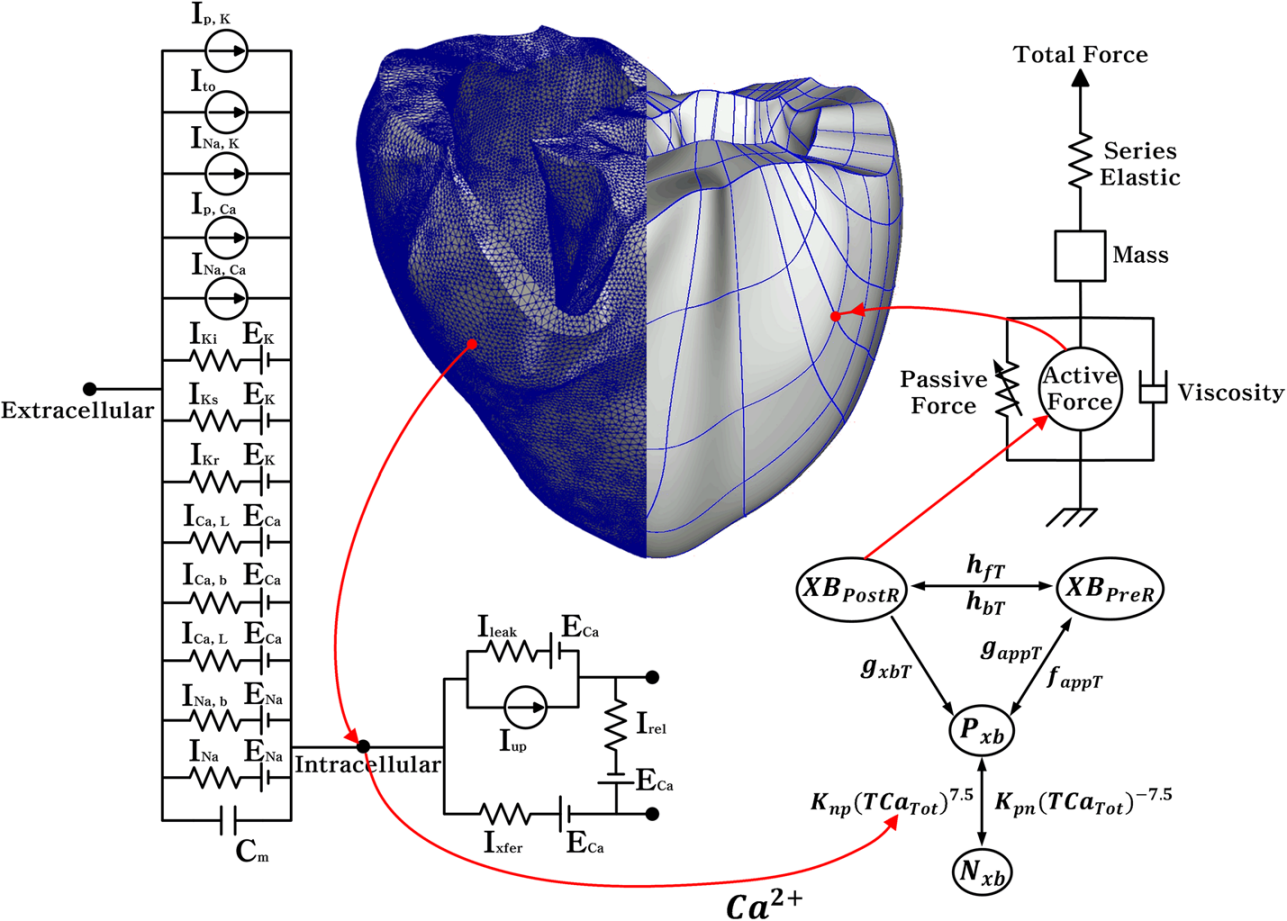
Schematic diagram of electromechanical simulation. The left side of the diagram is a ventricular model of electrophysiological simulation. The electrical components represent the currents, pumps, and ion exchangers from the Ten Tusscher ion model, which emulates the cell membrane for ion transport and the sarcoplasmic reticulum within cardiac cells. “I” represents the ion currents, and “E” is equilibrium potentials of each ion. (For more details, see the text.) The mechanical components on the right side are myofilament models proposed by Rice et al. [14]. *N*_*xb*_ and *P*_*xb*_ are nonpermissive and permissive confirmation of regulatory proteins, respectively. *K*_*np*_ and *K*_*pn*_ are transition rates, *K*_*np*_(*TCa*_*Tot*_)^7.5^ is the forward rate of the nonpermissive-to-permissive transition, and, working in the opposite direction, and *K*_*pn*_(*TCa*_*Tot*_)^−7.5^ is the backward rate of the permissive-to-nonpermissive transition. *g*_*xbT*_ is the ATP-consuming detachment transition rate, *h*_*fT*_ and *h*_*bT*_ are the forward transition rate and the backward transition rate, respectively; *f*_*aapT*_ is the crossbridge attachment rate of transition to the first strongly bound state *XB*_*PreR*_, and *g*_*aapT*_ is the reverse rate. *XB*_*PreR*_ represents prerotated states of the myosin head in relation to binding. *XB*_*PostR*_ denotes a strongly bound myosin head

The human ventricular model used in this study has two dynamic characteristics: electrophysiological conduction and mechanical contraction. The part of the model simulating electrophysiological characteristics consisted of a lumped parameter circuit to mimic the phenomenon of ion exchange through the cell membrane of a myocyte. In Fig. 1, I_p, K_ represents the current due to the K^+^ pump, I_to_ denotes the transient outward K^+^ current, I_Na, K_ is the current by the Na^+^–K^+^ ion exchange pump, I_p, Ca_ is the current of the sarcoplasmic Ca^2+^ pump, and I_Na, Ca_ is the current mediated by the Na^+^–Ca^2+^ ion exchange pump. E_K_, E_Ca_, and E_Na_ represent equilibrium potentials of K^+^, Ca^2+^, and Na^+^ ions, respectively, whereas C_m_ denotes the membrane capacitance due to the phospholipid bilayer in ventricular cells. I_Ki_ is the inward rectifier K^+^ current, I_Ks_ is the K^+^ current due to the slow delayed rectifier, I_Ca, L_ represents the L-type inward Ca^2+^ current, and I_Ca, b_ denotes the background Ca^2+^ current. I_Na, b_ represents the background Na^+^ current, and I_Na_ is the fast inward Na^+^ current. I_rel_ means the Ca^2+^ current that is released from the junctional sarcoplasmic reticulum (JSR), I_leak_ denotes the Ca^2+^ current that leaks from the JSR, and I_up_ is the Ca^2+^ uptake current into the network sarcoplasmic reticulum (NSR).

The part of the model simulating mechanical contraction characteristics was constructed to mimic cardiac contraction by means of the crossbridge of a myofilament. In Fig. 1, *XB*_*PreR*_ represents prerotated states of the myosin head in relation to binding and contributes to stiffness but does not generate force in the absence of net motion. *XB*_*PostR*_ denotes a strongly bound myosin head and represents the isomerization to induce strain in the extensible neck region. The force due to the crossbridge can be subdivided into an active force and a passive force. The active force induces the action of the cycling crossbridge, and the passive force induces the complete muscle response with viscoelastic elements. Mass prevents instantaneous changes in muscle shortening velocity for quick-release protocols, whereas a linear elastic element is intended to simulate the effects of compliant end connections that take place in real muscle preparations.

The electrophysiological part of the model is based on the ion model proposed by Ten Tusscher et al. [12]. This model, which reproduces the conduction phenomenon of APs in myocardial cells, was applied to the electrical conduction equation based on continuum mechanics:

1 $$\frac{{{\text{dV}}_{\text{m}} }}{\text{dt}} = - \frac{{{\text{I}}_{\text{ion}} + {\text{I}}_{\text{stim}} }}{{{\text{C}}_{\text{m}} }}$$

where V_m_ is the membrane potential, t is time,${\text{I}}_{\text{ion}}$ is the sum of all transmembrane ionic currents, I_stim_ is the current due to the external stimulus, and C_m_ is membrane capacitance.

To represent electrical propagation by means of conduction in three dimensional space, the partial differential equation expressing the electric conduction phenomenon in myocardial tissue and the ordinary differential equation for the electrical wave propagation of the ionic channel were derived [12–14].

2 $$\frac{{\text{dV}}_{\text{m}}}{\text{dt}} = - \frac{{\text{I}_{\text{ion}}} + {\text{I}_{\text{stim}}}}{\text{C}_{\text{m}}} +\frac{1}{\uprho_{\text{x}} {\text{S}_{\text{x}}} {\text{C}_{\text{m}}}} \frac{\partial^{2} {\text{V}}}{\partial {\text{x}}^{2} } +\frac{1}{\uprho_{\text{y}} {\text{S}_{\text{y}}} {\text{C}_{\text{m}}}} \frac{\partial^{2} {\text{V}}}{\partial {\text{y}}^{2} }+\frac{1}{\uprho_{\text{z}} {\text{S}_{\text{z}}} {\text{C}_{\text{m}}}} \frac{\partial^{2} {\text{V}}}{\partial {\text{z}}^{2} }$$

where ρ_x_, $\uprho_{\text{y}}$, and $\uprho_{\text{z}}$ denote cell resistance in x, y, and z directions, respectively. S_x_, ${\text{S}}_{\text{y}}$, and S_z_ represent the ratio of the volume to the surface in x, y, and z directions, respectively. In the model of Ten Tusscher et al. [12], I_ion_ was calculated as follows:

3 $$\begin{aligned} {\text{I}}_{\text{ion}} & = {\text{I}}_{\text{Na}} + {\text{I}}_{\text{Ki}} + {\text{I}}_{\text{to}} + {\text{I}}_{\text{Kr}} + {\text{I}}_{\text{Ks}} + {\text{I}}_{{{\text{Ca}},{\text{L}}}} + {\text{I}}_{{{\text{Na}}, {\text{Ca}}}} + {\text{I}}_{{{\text{Na}},{\text{K}}}} \\ & \quad + {\text{ I}}_{{{\text{p}},{\text{Ca}}}} + {\text{ I}}_{{{\text{p}},{\text{K}}}} + {\text{ I}}_{{{\text{b}},{\text{Ca}}}} + {\text{ I}}_{{{\text{b}},{\text{Na}}}} \\ \end{aligned}$$

where I_Na_ is the Na^+^ current. I_Ki_, I_to_, I_Kr_, and I_Ks_ respectively represent K^+^ currents: the inward rectifier K^+^ current, transient outward K^+^ current, rapid delayed rectifier K^+^ current, and slow delayed rectifier K^+^ current. I_Ca,L_ is the L-type inward Ca^2+^ current, I_Na,Ca_ is the Na^+^–Ca^2+^ exchange current, I_Na,K_ denotes the Na^+^–K^+^ exchange current,${\text{I}}_{{{\text{p}},{\text{Ca}}}}$ is the current of the Ca^2+^ pump, I_p,K_ represents the current of the K^+^ pump, I_b,Ca_ is the background Ca^2+^ current, and I_b,Na_ denotes the background Na^+^ current.

We used transient Ca^2+^ data from electrophysiological simulation for the electromechanical simulation. We employed Ten Tusscher’s equation to express the Ca^2+^ dynamics, which induce contraction of the thin filament via the Ca^2+^ induced Ca^2+^ released (CICR) current and generates tension.

4 $${\text{I}}_{\text{leak}} = {\text{V}}_{\text{leak}} \left( {{\text{Ca}}_{\text{sr}} - {\text{Ca}}_{\text{i}} } \right)$$

5 $${\text{I}}_{\text{up}} = \frac{{{\text{V}}_{\text{maxup}} }}{{1 + {\text{K}}_{\text{up}}^{2} /{\text{Ca}}_{\text{i}}^{2} }}$$

6 $${\text{I}}_{\text{rel}} = \left( {{\text{a}}_{\text{rel}} \frac{{{\text{Ca}}_{\text{sr}}^{2} }}{{{\text{b}}_{\text{rel}}^{2} + {\text{Ca}}_{\text{sr}}^{2} }} + {\text{c}}_{\text{rel}} } \right){\text{dg}}$$

7 $$\frac{{{\text{dCa}}_{\text{itotal}} }}{\text{dt}} = - \frac{{{\text{I}}_{{{\text{Ca}}, {\text{L}}}} + {\text{I}}_{{{\text{Ca}}, {\text{b}}}} + {\text{I}}_{{{\text{p}},{\text{Ca}}}} - 2{\text{I}}_{{{\text{Na}},{\text{Ca}}}} }}{{2{\text{V}}_{\text{C}} {\text{F}}}} + {\text{I}}_{\text{leak}} - {\text{I}}_{\text{up}} + {\text{I}}_{\text{rel}}$$

8 $$\frac{{{\text{dCa}}_{\text{srtotal}} }}{\text{dt}} = \frac{{{\text{V}}_{\text{c}} }}{{{\text{V}}_{\text{SR}} }}\left( { - {\text{I}}_{\text{leak}} + {\text{I}}_{\text{up}} - {\text{I}}_{\text{rel}} } \right)$$

where I_leak_, I_up_, and I_rel_ denote leakage current from the sarcoplasmic reticulum (SR) to the cytoplasm, pump current taking up calcium in SR, and CICR current, respectively. V_leak_ is maximal I_leak_, V_maxup_ is maximal I_up_. Ca_i-_ and Ca_sr_ represent the free calcium concentration in the cytoplasm and in the SR. K_up_ is the half-saturation constant of I_up_. a_rel_, b_rel_, and c_rel_ are maximal Ca_sr_-dependent I_rel_, Ca_sr_ half-saturation constant of I_rel_, and maximal Ca_sr_-independent I_rel_, respectively. d is the activation gate of I_rel_, and g is the calcium-dependent inactivation gate of I_rel_. Ca_itotal_ represents total Ca^2+^ in the cytoplasm, and Ca_srtotal_ is the total amount of Ca^2+^ in the SR. V_c_ and V_SR_ are the cytoplasm and SR volumes, respectively. F is the Faraday’s constant.

The Ca^2+^-binding mechanism in the canine cardiac muscle model proposed by Rice et al. was utilized here to describe the crossbridge, which means contraction in the sarcomere [15]. Thus, the length of the sarcomere (SL) affected by the crossbridge is expressed as follows:

9 $$\frac{\text{d}}{{\text{dt}}}{\text{SL}}= \frac{\int{Force} + \left( {\text{SL}}_{0} - {\text{SL}} \right) \times {\text{viscosity}}}{\text{mass}}$$

where Integral_Force_ represents the sum of the normalized forces integrated by time, and viscosity is the viscous factor in the crossbridge. This equation implies contraction or expansion of the sarcomere (not isosarcometric conditions). In the case of isosarcometric conditions, $\frac{\text{d}}{{\text{dt}}}{\text{SL}}= 0$, and SL takes initial value SL_0_.

10 $$\mathop \int \nolimits Force = \mathop \int \limits_{0}^{t} \left( {F_{active} \left( x \right) + F_{passive} \left( x \right) - F_{preload} - F_{afterload} \left( x \right)} \right){\text{dt}}$$

11 $$F_{afterload} \left( x \right) = KSE \times \left( {x - SL_{0} } \right)$$

where F_active_ (x) is defined as active force, and F_passive_ (x) represents passive force. The term F_preload_ is a constant force. As this would induce an initial sarcomere length that is larger than the resting length, F_preload_ corresponds to F_passive_ (SL_0_). F_afterload_ term is differently used for isotonic contraction and isometric contraction. Under the isotonic contraction condition, this term is fixed after the release. On the other hand, under the isometric contraction, this term is used to simulate compliant ends of the muscle as shown Eq. (11). Here, *x* is sarcomere length, and KSE denotes the stiffness in units of normalized force per μm.

### Three dimensional ventricular electromechanical model

To achieve our goal, we adapted an image-based electromechanical model of the human ventricular heart from Johns Hopkins University [16]. Human ventricular geometries were generated using the methodology described by Gurev et al. [17–20]. The three-dimensional human ventricular finite-element model used in this study consists of a lumped-parameter model of the physiological circulatory system [20, 21]. Our ventricular model for the electrophysiological simulation consisted of 214,319 tetrahedral finite elements. In contrast, the ventricular model for electromechanical simulation consisted of 14,720 Hermite-based finite elements to represent a natural three-dimensional curve of the cardiac surface [14, 22, 23]. Three dimensional simulation was carried out using a human arrhythmic ventricle model including the Purkinje fiber mesh, in which propagation of electrical stimuli during electrophysiological simulation starts from the atrioventricular node and spreads throughout the ventricle by means of the Purkinje networks based on Berenfeld et al. [24]. In addition, considering the differences in the structural characteristics and ventricle thickness, we assumed that all tissues of the ventricles were heterogeneous, and the conductance varied depending on the part of the ventricle.

### Simulation protocol

In this study, we observed APD variation in single-cell and three-dimensional ventricular models. First, we used a cellular electrophysiological simulation to observe the electrical changes due to APD variation and to clarify the relationship between APD and calcium concentration. Second, we used a three-dimensional electromechanical simulation to compare cardiac pumping efficiency quantitatively under sinus rhythm conditions.

The single-cell simulation was performed using the ventricular cell model suggested by Ten Tusscher et al. [12]. The formulation of the I_Ks_ current equation was modified to examine the variation with the change in APD.

12 $${\text{I}_{\text{Ks}}} = {\text{g}_{\text{Ks}}} {\text{x}_{\text{s}}^{2}} \left( {{\text{V} - {\text{E}}}_{\text{Ks} }} \right)$$

where g_Ks_ is conductance of the K^+^ channel, x_s_ is the activated gate parameter of the K^+^ channel, and E_Ks_ denotes the equilibrium potential of K^+^. In this study, the initial values of g_Ks_ were set to 0.392 × 1.3 mS/μF. This value is based on other studies [12, 25]. Next, it was simulated by increasing the initial values 2-, 4-, 6-, 8-, and 10-fold to induce changes in APD.

The three dimensional ventricular simulation was conducted by setting the basic cycle length (BCL) to 600 ms. The change in APD during 1 cycle and the conduction wavelength were measured.

13 $${\text{Wavelength}} = {\text{CV }} \times {\text{APD}}_{90}$$

14 $${\text{CV }}\left( {{\text{cm}}/{\text{s}}} \right) = \frac{{{\text{Distance}}\left( {\text{cm}} \right)}}{{{\text{up}}\left( {\text{ms}} \right) - {\text{down}}\left( {\text{ms}} \right)}} \times 1000$$

where CV is the conduction velocity in myocardial cells. *APD*_90_ means the time point where ventricular cells become excited and 90% repolarized. For calculation of conduction velocity, we specified the node (down) located on the lower side of the ventricular model surface and the node (up) on the upper side of its vertically positioned surface. The conduction velocity was calculated by dividing the distance along the straight line between these two nodes by the difference in the time of the response to a stimulus between the two nodes.

Three-dimensional electromechanical simulations used data on transient Ca^2+^ concentration from electrophysiological simulation results. The contraction of the ventricles was caused by active tension in the dynamic model of a myosin filament proposed by Rice et al. [15]. To numerical APD on cardiac pumping efficacy, we compared changes in stroke volume, ejection fraction, stroke work, and ATP consumption rate for the six conditions (including the normal group) according to APD variation. Each value was measured in only the last cycle (600 ms) of the simulation to observe changes in the steady state [26, 27].

## Results

### Cellular electrophysiological responses

**Fig. 2:**
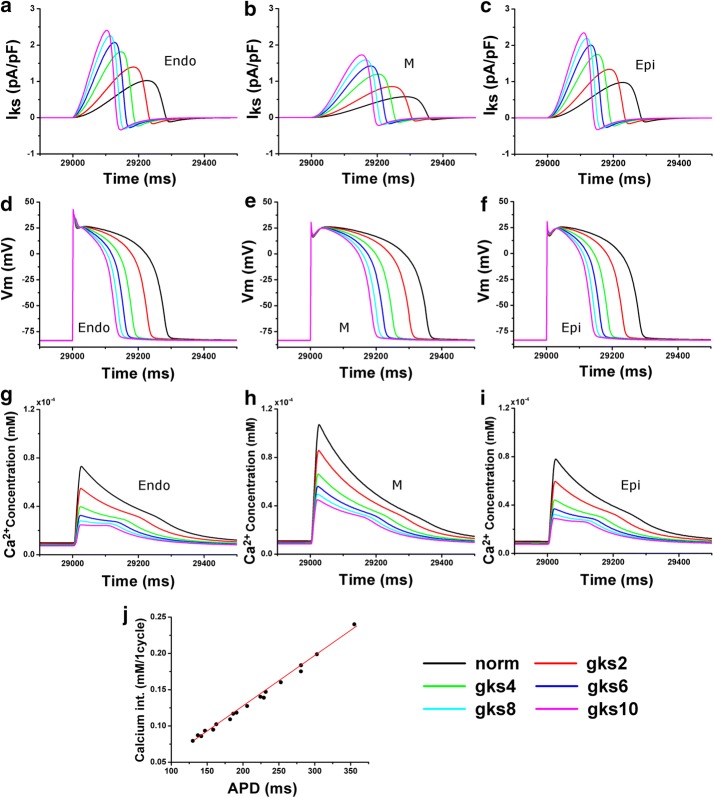
Cellular simulation results for ventricular tissue according to electrical conductivity conditions of the I_Ks_ channel. **a**–**c** I_Ks_ graphs as a function of electrical conductivity *g*_*Ks*_ of the I_Ks_ channel. **d**–**f** Action potential shape under the influence of variation of electrical conductivity. **g**–**i** Ca^2+^ concentration. **j** Ca^2+^ integration depending on APD variation. “norm” indicates the case where *g*_*ks*_ has a normal value. ‘gks2’, ‘gks4’, ‘gks6’, ‘gks8’, and ‘gks10’ represent electrical conductivity when *g*_*ks*_ is increased 2-, 4-, 6-, 8-, and 10-fold, respectively. ‘Endo’ is the endocardial cell, ‘M’ is the mid-myocardial cell, and ‘Epi’ is the epicardial cell. ‘APD’ denotes action potential duration

We performed a single cellular electrophysiological simulation by increasing electrical conductivity of the I_Ks_ channel, which affects the APD of myocardial cells. The results are presented in Fig. 2.

Figure 2a–c shows intensity of the I_Ks_ current depending on the electrical conductivity of the I_Ks_ channel, where A is an “endocardium cell,” B is a “midmyocardium cell,” and C is an “epicardium cell.” In each graph, “normal” is the case where the electrical conductivity of the I_Ks_ channel is normal. Labels “gks2” to “gks10” indicate that the electrical conductivity of the I_Ks_ channel is 2, 4, 6, 8, or 10 times the normal value, respectively. In Fig. 2a–c, the current flowing through the I_Ks_ channel is increased due to the increased electrical conductivity of the I_Ks_ channel. The I_Ks_ current, which was slowly delayed at normal electrical conductivity, was rapidly delayed as the electrical conductivity of the I_Ks_ channel increased.

In the three ventricular tissues, *APD*_90_ shortened as the electrical conductivity of the I_Ks_ channel increased (Fig. 2d–f). In the results of ventricular cellular simulation, the *APD*_90_ measured in an endocardium cell was 279 ms when the electrical conductivity of the I_Ks_ channel was normal. *APD*_90_ was 228 ms when the electrical conductivity was doubled, 182 ms when it was quadrupled, 158 ms when multiplied by 6, 141 ms when multiplied by 8, and 123 ms when multiplied by 10. The *APD*_90_ measured in a midmyocardium cell was 353, 301, 249, 222, 202, and 189 ms, corresponding to electrical conductivity of the I_Ks_ channel when it was normal or increased 2-, 4-, 6-, 8-, and 10-fold. The *APD*_90_ measured in an epicardium cell was 280, 230, 185, 162, 147, and 135 ms, corresponding to the electrical conductivity of the I_Ks_ channel when it was normal or increased 2-, 4-, 6-, 8-, and 10-fold. *APD*_90_ is the highest in the midmyocardium cell compared to the other two cell types in all cases owing to the change in electrical conductivity of the I_Ks_ channel.

Figure 2g–i show a graph of the intracellular Ca^2+^ concentration as a function of electrical conductivity of the I_Ks_ channel in the ventricular tissue during a BCL of 600 ms. As the electrical conductivity of the I_Ks_ channel increased in all three ventricular cell types, the wavelength of the Ca^2+^ concentration graph was almost constant, but the amplitude significantly diminished. The amplitude of Ca^2+^ in the normal case was ~ $0.8 \times 10^{ - 4}$ mM in the endocardium and epicardium cells (Fig. 2g and i). When the conductivity of the I_Ks_ channel was increased tenfold, the concentration was $0.2 \times 10^{ - 4}$ to $0.3 \times 10^{ - 4}$ mM. This result is similar to the data from the midmyocardium cell; the amplitude of the Ca^2+^ concentration graph in the normal case was $1.1 \times 10^{ - 4}$ mM, but the value decreased as the electrical conductivity of the I_Ks_ channel increased. When the electrical conductivity increased tenfold, Ca^2+^ concentration decreased to ~ $0.45 \times 10^{ - 4}$ mM.

Figure 2j showed the integration of Fig. 2d–i, which is the changes of intracellular calcium concentration in ventricular cells due to APD variation according to the change of electrical conductivity of the I_Ks_ channel during a 1-cycle period. The shortened APD lower the intracellular calcium concentration than normal group. There was a linear relationship between APD_90_ and intracellular calcium concentration in each ventricular cell.

### Three dimensional ventricular electrophysiological responses

**Fig. 3:**
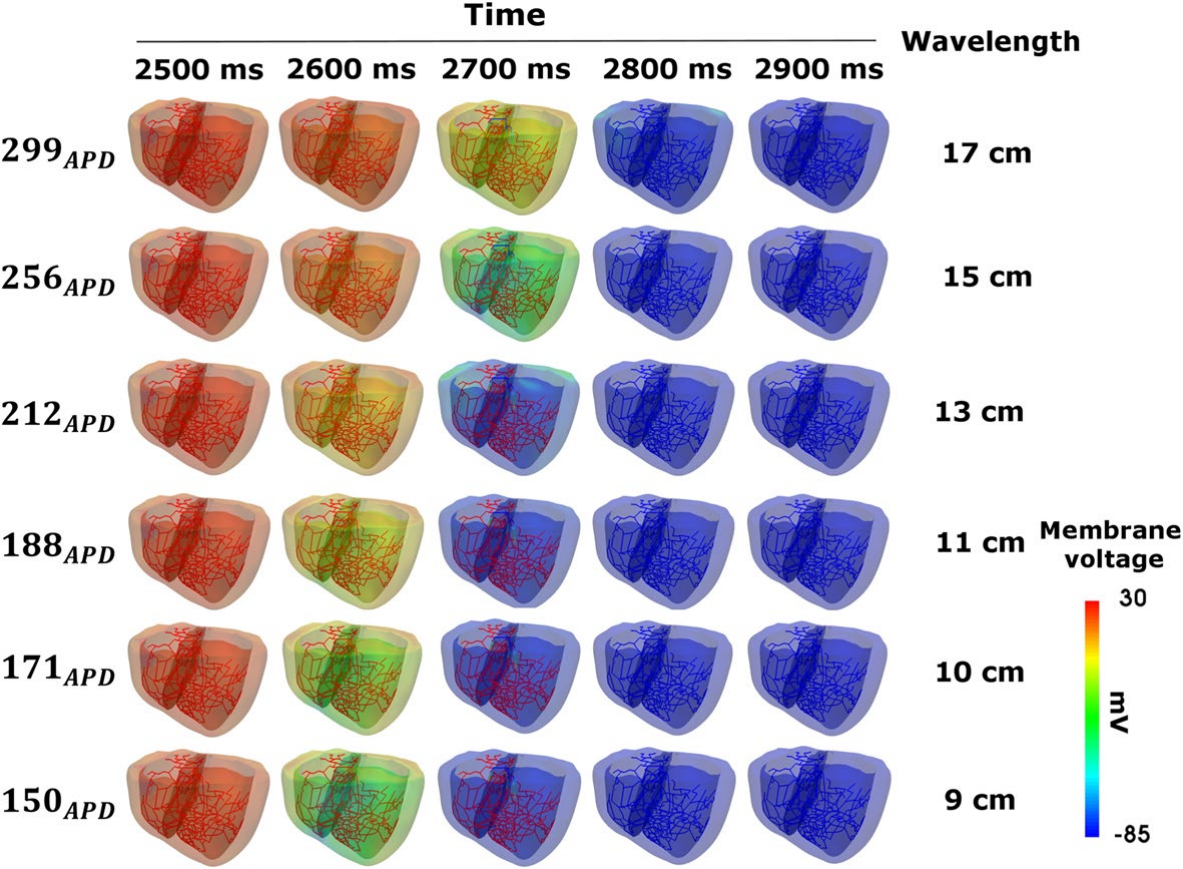
Snapshots and conduction wavelengths for three-dimensional electrophysiological simulations with APD variation. ‘APD’ denotes action potential duration

Figure 3 shows the electrophysiological simulation results from the three dimensional ventricular model. Simulation results during the BCL of 600 ms are snapshots taken at intervals of 100 ms on the horizontal axis, as a function of the variation of *APD*_90_ on the vertical axis. The vertical axis of Fig. 3 indicates that the *APD*_90_ measured by means of the three dimensional electrophysiological model was 299 ms (normal), 256 ms (gks2), 212 ms (gks4), 188 ms (gks6), 171 ms (gks8), and 150 ms (gks10). As *APD*_90_ decreased, the excitement resulting from the electrical stimulation delivered to ventricular tissue by the Purkinje networks was terminated faster. In the ventricular tissue, the conduction wavelength was 17 cm when *APD*_90_ was 299 ms, but it decreased as *APD*_90_ was shortened under the heterogeneous condition. When *APD*_90_ was 150 ms, the conduction wavelength became 9 cm by decreasing to 44% compared to normal group.

**Fig. 4:**
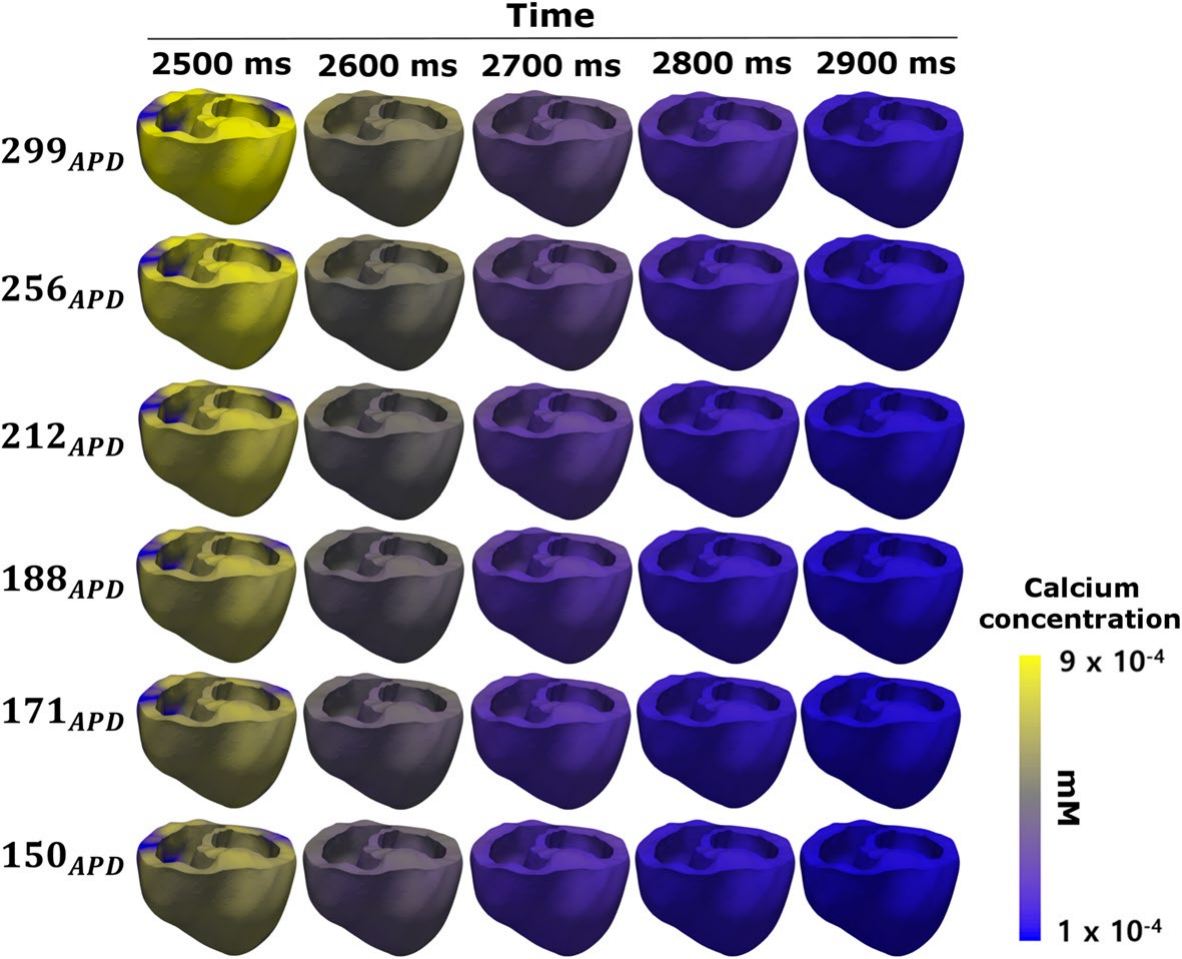
Calcium contour according to three-dimensional electrophysiological simulations with APD variation. ‘APD’ denotes action potential duration

Figure 4 showed the calcium contour due to APD variation in three dimensional electrophysiological simulations. As *APD*_90_ decreased, the period of Ca^2+^ concentration change on the ventricular surface at BCL 600 ms stayed almost the same. In contrast, in accordance with the shortening of *APD*_90_, a color change of the Ca^2+^ contour occurred at the time point 2500 ms. Although the Ca^2+^ contour at 2500 ms appears as the brightest yellow for 299_*APD*_, at 150_*APD*_, it was almost gray.

### Three dimensional ventricular mechanical responses

**Fig. 5:**
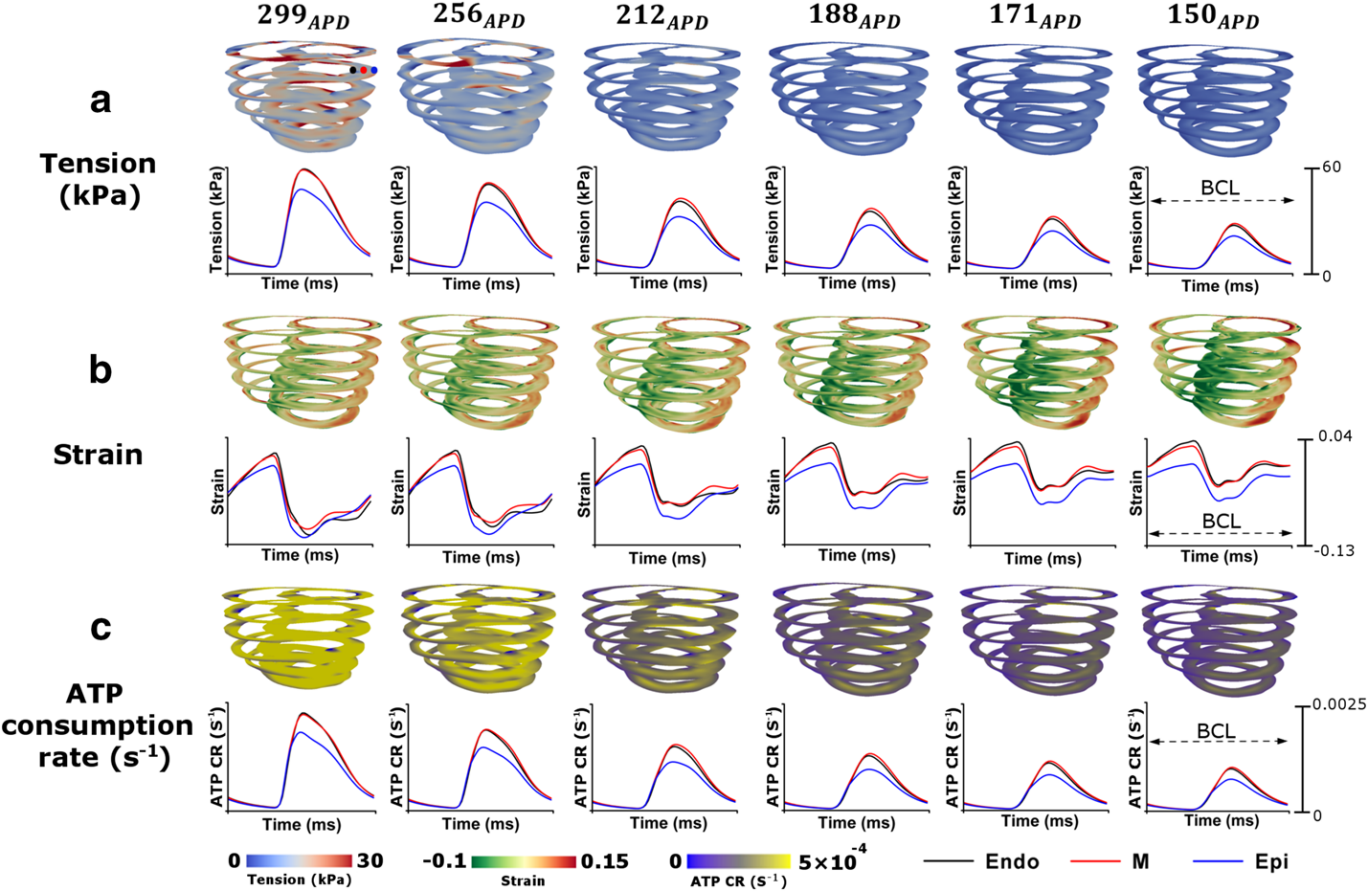
Changes in tension, strain, and ATP consumption rate depending on APD variation. Graphs showing variation in tension (**a**), strain (**b**) and ATP consumption rate (**c**) with time in each ventricular tissue: the endocardium (Endo), mid-myocardium (M), and epicardium (Epi). Snapshots of end-systolic tension, ATP consumption rate, and end-diastolic strain in a three-dimensional sinus pacing simulation. ‘ATP CR’ denotes ATP consumption rate, and ‘BCL’ denotes basic cycle length, which is 600 ms

The effects of APD variation on the pumping efficiency depending on the electrical conductivity of the I_Ks_ channel were analyzed next. Figure 5 depicts a three-dimensional ventricular contour that shows the muscle tension, adenosine triphosphate (ATP) status at the end-systolic volume (ESV) time point, strain at the end-diastolic volume (EDV) time point, and a graph of each variable (tension, strain, and ATP) at BCL 600 ms by ventricular cell type.

The shortening of *APD*_90_ decreased the contraction force of the muscle at the ESV time point and increased the strain of the ventricles at the EDV time point. In addition, ATP consumption decreased in ventricular cells at the ESV time point.

The total consumption of ATP at the ESV time point was ~ 136 s^−1^ at 299_*APD*_, and the average consumption was ~ 0.000594 s^−1^. In this case, total tension was 3,468,332 kPa and contracted to an average of 15.15 kPa. At 256_*APD*_, the total amount of ATP consumed at the ESV time point was ~ 95 s^−1^, and the average was 0.000414 s^−1^. End systolic tension at *APD*_90_ of 256 ms was 2,350,950 kPa in total and decreased to an average of 10.27 kPa The total consumption and the average of ATP at 212_*APD*_ were found to be 64.9 and 0.000284 s^−1^, respectively. The corresponding total tension was 1,533,506 kPa, and the average was 6.7 kPa. Similarly, the total consumption and average ATP consumption at 188_*APD*_, 171_*APD*_, and 150_*APD*_ were 53.2 and 0.000232 s^−1^ (188_*APD*_), 45.2 and 0.000198 s^−1^ (171_*APD*_), and 42.9 and 0.000187 s^−1^ (150_*APD*_), respectively. The total amount of and average tension in each case were calculated and found to be 1,211,614 and 5.29 kPa (188_*APD*_), 992,201 kPa and 4.33 kPa (171_*APD*_), and 927,608 and 5.05 kPa (150_*APD*_), respectively.

In addition, during 600 ms, the difference in the change of ATP and tension was noticeable due to shortened APD in each ventricular tissue. The amplitude of the tension change in the endocardium and midmyocardium at 299_*APD*_ was ~ 60 kPa, but at 150_*APD*_, it was ~ 30 kPa. The tension graph of the epicardium shows a smaller amplitude in all cases depending on APD variation in comparison with the other two ventricular tissues. These results were also obtained from the graph of ATP consumption by ventricular tissue. The graph of amplitude of ATP consumption in the endocardium and midmyocardium at 299_*APD*_ shows ~ 0.0025 s^−1^, but the amplitude in the epicardium was smaller. The amplitude of the ATP graph was smaller as *APD*_90_ decreased, and the change in ATP consumption at 150_*APD*_ during BCL of 600 ms was reduced by 50% as compared to 299_*APD*_.

On the other hand, the degree of deformation of the ventricles during BCL of 600 ms decreased as *APD*_90_ was shortened, and the graph of the strain change shifted upward. The strain variable was smaller in the epicardium than in the other ventricular tissues. As *APD*_90_ decreased, the difference in strain graphs between the two ventricular tissues and the epicardium increased.

**Fig. 6:**
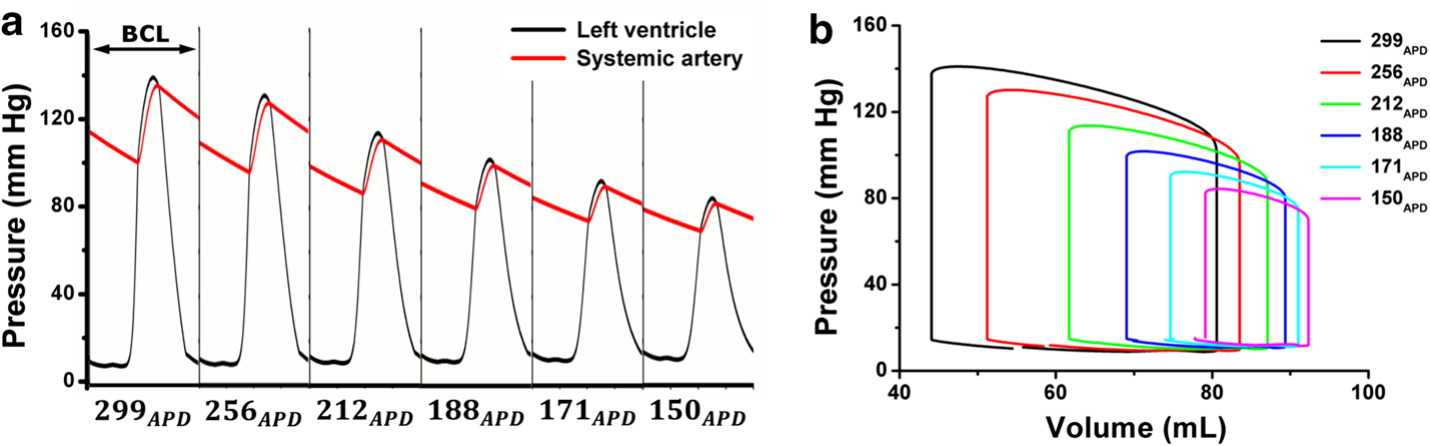
Mechanical-response graphs for the electromechanical simulation involving the three dimensional ventricular-tissue model with APD variation. **a** Pressure in the left ventricle and systemic arteries. **b** Pressure–volume loop (PV loop) of the left ventricle. ‘BCL’ is basic cycle length, which is 600 ms

**Fig. 7:**
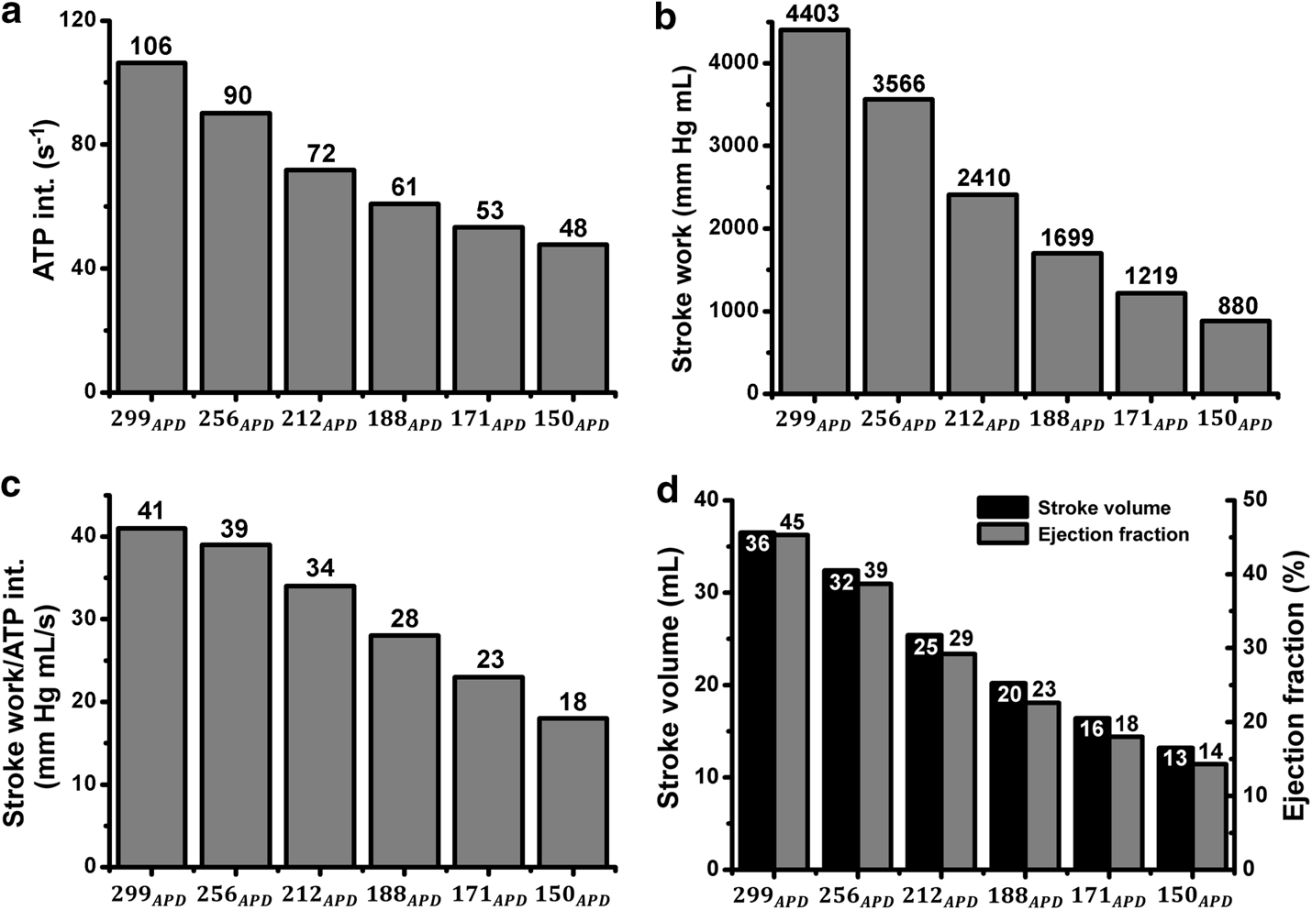
APD-dependent cardiac pumping efficacy changes in electromechanical simulation using a 3D ventricular-tissue model. **a** Energy consumption of the left ventricle in the form of ATP. **b** Stroke work of the left ventricle. **c** Rate of stroke work for ATP consumption. Amount of work done by the left ventricle relative to energy consumption. **d** Stroke volume-ejection fraction for the left ventricle

Analysis of the changes in the mechanical responses and cardiac pumping efficiency of the ventricle depending on APD variation is shown in Figs. 6, 7. Figure 6 depicts a dynamic response graph of the three dimensional electromechanical simulation. The pressure in the left ventricle and in systemic arteries decreased for the sinus rhythm. EDV and ESV increased due to shortening of *APD*_90_. The pressure–volume (PV) loop was shifted to the left as *APD*_90_ was shortened (Fig. 6a, b). As a result, the pulse pressure at 299_*APD*_ was 35.1 mmHg, but the value decreased with the shortening of *APD*_90_. In each case, the pulse pressure was 30.7 mmHg (256_*APD*_), 24.2 mmHg (212_*APD*_), 19.3 mmHg (188_*APD*_), 15.3 mmHg (171_*APD*_), and 12.2 mmHg (150_*APD*_).

EDV is the maximal volume, and ESV is the minimal volume of the PV loop in Fig. 6b. At 299_*APD*_, EDV of the left ventricle was 80.6 mL, and ESV was 44.1 mL. On the other hand, EDV and ESV were 92.3 and 79.1 mL, respectively, at 150_*APD*_. As *APD*_90_ decreased, the pressure in the left ventricle diminished, and the volume increased. Accordingly, the internal area of the PV loop (which moved to the left in each case) decreased. EDV and ESV were 83.6 and 51.2 mL (256_*APD*_), 87.1 and 61.7 mL (212_*APD*_), 89.2 and 69 mL (188_*APD*_), and 91 and 74.6 mL (171_*APD*_), respectively.

Figure 7 shows changes in the cardiac pumping efficiency depending on APD variation in the three dimensional ventricular-tissue model. Figure 7a depicts quantification of total ATP consumed to produce contraction from the crossbridge of a myofilament during BCL of 600 ms, and its value decreased as *APD*_90_ was shortened. In addition, the stroke work in the ventricle decreased for 1 cycle. Its value, which was 4403 mmHg mL at 299_*APD*_, decreased by ~ 80% at 880 mmHg mL (150_*APD*_; Fig. 7b). It was computed from the internal area of the PV loop in Fig. 6b.

This change reduced the amount of ventricular work per unit ATP consumed by the myofilament during the sinus rhythm in accordance with shortened *APD*_90_ (Fig. 7c). This means that as *APD*_90_ is shortened, energy efficiency of the ventricle during 1 cycle decreased. The energy efficiency at 299_*APD*_ was 41, and the efficiency at 39, 34, 28, and 23 is respectively shown at 256_*APD*_, 212_*APD*_, 188_*APD*_, and 171_*APD*_. At 150_*APD*_, this efficiency was the lowest: 18.

Figure 7d is a graph of stroke volume and the ejection fraction calculated from EDV to ESV in the PV loop of Fig. 6b. The stroke volume was measured by means of the difference between EDV and ESV in the PV loop, and the ejection fraction was obtained by means of the stroke volume ratio for EDV. These data refer to the amount and efficiency of blood that is sent from the heart to the aorta and the pulmonary artery. Stroke volume and ejection fractions at 299_*APD*_ were measured and found to be 36 mL and 45%, respectively. Nonetheless, these values decreased with decreasing *APD*_90_, resulting in 13 mL and 14% at 150_*APD*_. The stroke volume and the ejection fraction of the left ventricle decreased as *APD*_90_ was shortened. The pumping efficiency when *APD*_90_ became 150 ms was 68% lower relative to the value at 299_*APD*_.

## Discussion

In this simulation study, we analyzed the ventricular pacing efficiency under APD variation. The main findings of the study are the following:

1. In the cellular electrophysiological simulation, as APD is shortened owing to the increase in the electrical conductivity of the K^+^ channel, the intracellular Ca^2+^ concentration decreases. That is, the APD and the sum of the intracellular Ca^2+^ concentrations showed a positive correlation.
2. The shortened APD reduced the conduction wavelength in the three-dimensional ventricular tissue by shortening the plateau and early repolarization in myocardial cells.
3. In addition, the shortened APD reduced cardiac pumping efficiency by more than 60% compared with the normal group.

Increasing the electrical conductivity (*g*_*Ks*_) of the I_Ks_ channel makes the flow of I_Ks_ currents faster owing to K^+^ flowing out of the cells. The fast I_Ks_ current induces rapid repolarization of the APs, leading to rapid return to the resting cell membrane potential. This event reduces *APD*_90_ and shortens the opening time of the L-type Ca^2+^ channel. Finally, because the period for extracellular Ca^2+^ to flow into the intracellular area decreases, the amount of Ca^2+^ entering the cell during one cycle decreases. That is, as APD decreases, the concentration of Ca^2+^ in the cells decreases. This finding is consistent with the result of Ten Tusscher et al. [12, 28]. From these results, we can conclude that there is a linear relationship between APD and intracellular Ca^2+^ concentration within the range of our experimental conditions (Fig. 2j).

In addition, cardiac tissues composed of cells with shortened APD have short wavelengths even if the conduction velocity is the same (Fig. 3). These results are consistent with those of Roden et al. [6]: in cell types with shortened APD, the period of return to the resting phase is reduced, even though the whole tissue is excited at the same time in one cycle of the sinus rhythm [29]. This finding indicates that the cells with short APD decrease the depolarization period, thereby reducing the opening period of the voltage-dependent L-type Ca^2+^ channel and reducing the intracellular Ca^2+^ concentration (Fig. 4).

Intracellular Ca^2+^ includes Ca^2+^ ions that are stored in the SR and released into the cells (the Ca^2+^-induced Ca^2+^-released current). Ca^2+^ released into the cell binds to troponin, causing a conformational change in tropomyosin and formation of the crossbridge where the myosin head binds to the actin filament. In other words, the strength of the tension generated during the crossbridge of the myofilament is proportional to the concentration of Ca^2+^ in the cell. However, in tissues with short APD, the amount of Ca^2+^ released into the cells from the SR is lower. Therefore, the Ca^2+^ concentration in the cells decreases, and the strength of the myocardial tension diminishes (Figs. 4 and 5a). This state weakens the contraction force of the ventricular muscle, thereby reducing heart rate and inhibiting blood circulation (Fig. 6a, b) [30, 31]. In addition, the end-diastolic strain of the ventricle increases as the contraction force decreases (Fig. 5b), and the amount of ATP consumed in the ventricle during one cycle decreases as the crossbridge generation diminishes under conditions of short APD (Fig. 5c) [29, 32].

Therefore, in the tissue with shortened APD, the strength of the tension is low because of the low Ca^2+^ concentration, which reduces the myocardial contractility, thus reducing the stroke work produced by the ventricle during one cardiac cycle (Fig. 7b) [29]. Additionally, myocardial contractility weakened due to a reduction in tension also results in a decrease in the stroke volume of the ventricle (Fig. 7d). The decline in the stroke volume, which is the volume of blood pumped from the left ventricle per beat, leads to a decrease in the pressure in the left ventricle and lowered aortic pressure (blood pressure) according to Poiseuille’s law (Fig. 6a) [26, 31, 33, 34]. Thus, the amount of work performed by the ventricle, which is the ratio of stroke work to ATP consumption, is lower than that of a ventricular tissue with normal APD (Fig. 7b). The pumping efficiency of the ventricles can be deduced from the ratio of cardiac work rate to energy consumption [32, 33, 35, 36]. These results suggest that ventricular pacing efficiency is lower in tissues with shortened APD than in tissues with normal APD (Fig. 7c, d).

## Conclusions

The shortening of APD owing to increased electrical conductivity of a protein channel on myocardial cells likely decreases the wavelength and the pumping efficiency of the ventricles. Additionally, it may increase tissue sensitivity to ventricular fibrillation, including reentry, and cause symptoms such as dyspnea and dizziness.
